# Novel Gastric Cancer Stem Cell-Related Marker LINGO2 Is Associated with Cancer Cell Phenotype and Patient Outcome

**DOI:** 10.3390/ijms20030555

**Published:** 2019-01-28

**Authors:** Jung Hyun Jo, Soo Been Park, Semi Park, Hee Seung Lee, Chanyang Kim, Dawoon E. Jung, Si Young Song

**Affiliations:** 1 Division of Gastroenterology, Department of Internal Medicine, Yonsei University College of Medicine, Seoul 03722, Korea; JUNGHYUNJO83@yuhs.ac (J.H.J.); ELLIE0813@yuhs.ac (S.B.P.); LHS6865@yuhs.ac (H.S.L.); MISSKCY90@yuhs.ac (C.K.); 2 Department of Internal Medicine, Graduate School, Yonsei University College of Medicine, Seoul 03722, Korea; semi2010.park@samsung.com; 3 Center for Health Promotion, Samsung Medical Center, Sungkyunkwan University School of Medicine, Seoul 06351, Korea; 4 Institute of Gastroenterology, Yonsei University College of Medicine, Seoul 03722, Korea

**Keywords:** LINGO2, gastric cancer, cancer stem cells, epithelial to mesenchymal transition, tumorigenesis, angiogenesis, and tissue microarray analysis

## Abstract

The expression of leucine-rich repeat and immunoglobulin-like domain-containing nogo receptor-interacting protein 2 (LINGO2) has been reported in Parkinson’s disease; however, its role in other diseases is unknown. Gastric cancer is the second leading cause of cancer death. Cancer stem cells (CSC) are a subpopulation of cancer cells that contribute to the initiation and invasion of cancer. We identified LINGO2 as a CSC-associated protein in gastric cancers both in vitro and in patient-derived tissues. We studied the effect of LINGO2 on cell motility, stemness, tumorigenicity, and angiogenic capacity using cells sorted based on LINGO2 expression and LINGO2-silenced cells. Tissue microarray analysis showed that LINGO2 expression was significantly elevated in advanced gastric cancers. The overall survival of patients expressing high LINGO2 was significantly shorter than that of patients with low LINGO2. Cells expressing high LINGO2 showed elevated cell motility, angiogenic capacity, and tumorigenicity, while LINGO2 silencing reversed these properties. Silencing LINGO2 reduced kinase B (AKT)/extracellular signal-regulated kinase (ERK)/ERK kinase (MEK) phosphorylation and decreased epithelial-mesenchymal transition (EMT)-associated markers—N-Cadherin and Vimentin and stemness-associated markers— POU class 5 homeobox 1 (OCT4) and Indian hedgehog (IHH), and markedly decreased the CD44^+^ population. These indicate the involvement of LINGO2 in gastric cancer initiation and progression by altering cell motility, stemness, and tumorigenicity, suggesting LINGO2 as a putative target for gastric cancer treatment.

## 1. Introduction

Gastric cancer is the fourth most common malignant tumor worldwide and the second leading cause of cancer death [1]. Although gastric cancer mortality has declined recently due to early diagnosis by endoscopy, its prognosis is still poor, and has a 5-year survival rate of 20% in most parts of the world, except in Korea and Japan [2]. The 5-year survival rate of gastric cancer is about 70% in Korea and Japan; this may be due to the effectiveness of the mass screening programs [3,4].

Cancer stem cells (CSCs) are a subpopulation of cancer cells that have a high self-renewal capacity within the tumors. Typically, the CSCs constitute less than 5% of total tumor cells and are critical in cancer initiation, invasion, metastasis, and drug resistance [5,6,7]. Recent studies have shown that cancer cells undergoing epithelial-mesenchymal transition (EMT) share many properties with CSCs [8,9,10]. Epithelial cells are tightly associated with neighboring cells through E-cadherin-containing adherent junctions. EMT is the process by which epithelial cells undergo phenotypic changes such as loss of cell-cell adhesion, loss of cellular polarity and enhanced migration and invasion capacity to become mesenchymal-like cells [11]. Aberrant activation of EMT disturbs normal epithelial homeostasis, inducing pathologic alterations such as fibrosis, cancer cell invasion, and metastasis to distant secondary sites. Thus, EMT is thought to be the initial and most important step in the cancer metastasis cascade [12,13,14]. Gastric CSCs were first reported by Takaishi et al. in experiments showing that CD44-positive gastric cancer cells have stem cell-like properties and showed an increased resistance to traditional chemotherapies and radiotherapies [15]. Moreover, CD44 expression was significantly associated with the expression of EMT-related molecules E-cadherin, Vimentin, Snail-1, and ZEB-1 in gastric cancer patients [16].

Using microarray analysis, we identified leucine-rich repeat and immunoglobulin-like domain-containing nogo receptor-interacting protein 2 precursor (LINGO2) as a novel gastric CSC-related marker. LINGO2 gene is located on chromosome 9 at 9p21.2. This membrane protein is 606 amino acids long and weighs 68 kDa. LINGO2 is a human paralog of LINGO1, with 61% sequence identity [17]. LINGO1 and LINGO2 are associated with essential tremor and Parkinson’s disease; however, the function of LINGO2 in carcinogenesis is unknown [18,19].

In our studies, we explored the role of LINGO2 as a regulator of cell motility and stemness in gastric cancer cells. LINGO2 expression was significantly increased in patients with advanced gastric cancer. Moreover, the overall survival of patients with strong LINGO2 expression was significantly shorter than that of patients with weak LINGO2 expression. Cells that highly expressed LINGO2 showed increased tumorigenicity and angiogenic capacity compared to cells with low LINGO2 expression. In addition, suppressing LINGO2 expression by shRNA decreased cell motility and down-regulated CSC-related markers. Our data suggest that LINGO2 is a new therapeutic target for gastric cancer which targets EMT as well as CSC.

## 2. Results

### 2.1. LINGO2 is Differentially Expressed in Gastric Cancer Sphere Cells

**Figure 1:**
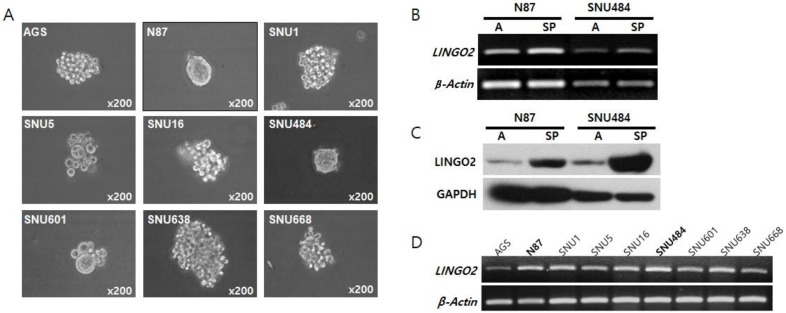
Expression of LINGO2 in gastric cancer cell-spheres (**A**) Gastric cancer cell lines including AGS, N87, SNU1, SNU5, SNU16, SNU484, SNU601, SNU638, and SNU668 were maintained with growth media and plated onto culture dishes or ultra-low attached plates in sphere media for sphere-formation assays and N87 and SNU 484 formed spheres. (**B**) LINGO2 mRNA expression and (**C**) protein expression was analyzed by RT-PCR and western blot, respectively, and LINGO2 mRNA as well as protein expression was elevated in gastric cancer sphere cells. (A, adherent cells; SP, sphere cells) (**D**) LINGO2 expression in all gastric cancer cell lines was analyzed by PCR analysis.

The sphere-forming capacity of different human gastric cancer cell lines including AGS, N87, SNU1, SNU5, SNU16, SNU484, SNU601, SNU638, and SNU668 was determined. N87 and SNU484 reproducibly formed spheres in vitro whereas other cell lines did not form spheres and remained as aggregated cell clusters (Figure 1A). The expression of stemness-related genes including jagged canonical notch ligand 2 (*JAG2*), notch receptor 3 (*NOTCH3*), hes family bHLH transcription factor 1 (*HES1*), Indian hedgehog (*IHH*), smoothened, frizzled class receptor (*SMO*), GLI family zinc finger 1 (*GLI1*), frizzled class receptor 1 (*FZD7*), *β-Catenin*, Nanog homeobox (*NANOG*), POU class 5 homeobox 1 (*OCT4*) and phosphatase and tensin homolog (*PTEN*) were elevated in spheres as determined by polymerase chain reaction (PCR) analysis and was further confirmed by microarray analysis (Supplementary Figure S1 and Supplementary Figure S2A). Among the differentially expressed genes, we focused on membranous as well as secretory proteins and observed that LINGO2 was up-regulated by 1.31-folds in N87 cells and 2.88-folds in SNU484 cells (Supplementary Figure S1 and Supplementary Table S1). We confirmed the elevated mRNA and protein expression of LINGO2 by PCR and western blot analysis, respectively (Figure 1B,C). Treatment with an anti-LINGO2 antibody decreased sphere numbers and sizes in both N87 and SNU484 cells (Supplementary Figure S2B). LINGO2 was constitutively expressed in all gastric cell lines with differences in basal expression levels (Figure 1D).

### 2.2. Increase in LINGO2 Expression Elevates Cancer Stem Cell Characteristics

**Figure 2:**
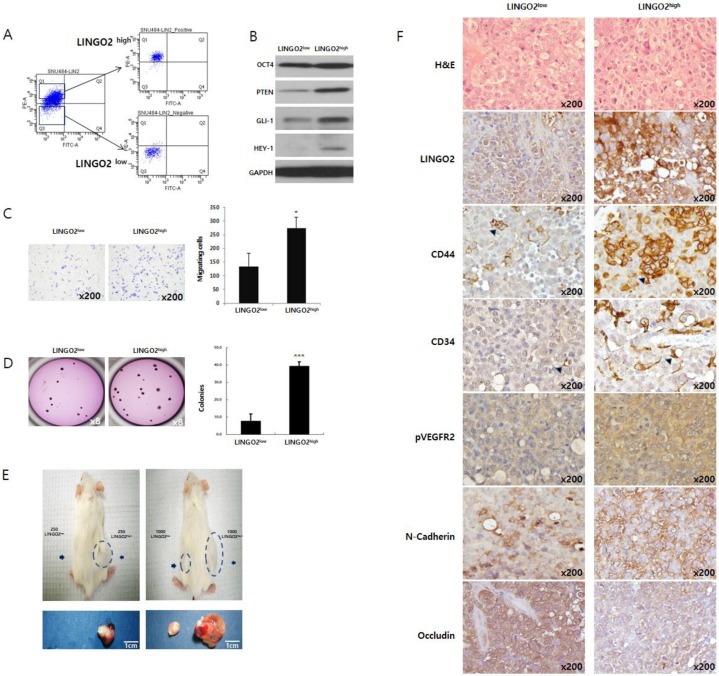
Cells highly expressing LINGO2 possess cancer stem cell characteristics. (**A**) Based on surface LINGO2 expression, SNU484 cells were sorted into LINGO2^high^ and LINGO2 ^low^ cells. (**B**) Elevated expression of cancer stem cells associated genes including OCT4, PTEN, Gli-1, and Hey-1 was observed in LINGO2^high^ cells than in LINGO2^low^ cells. (**C**) Cell migration increased by approximately 2-fold and (**D**) clonogenic ability increased by approximately 4-fold in LINGO2^high^ cells than in LINGO2^low^ cells (* *p* < 0.1, *** *p* < 0.001). Tumours are indicated by the dotted lines and arrows. (**E**) To assess the minimal number cells required for tumorigenesis, cells were subcutaneously injected into NOD/SCID mouse (*n* = 3 per group). LINGO2^high^ cells formed tumor mass with 250 cells whereas LINGO2^low^ started to form tumor with 1000 cells and more. Arrows indicated. (**F**) Immunohistochemical analysis of mouse tumor tissues revealed up-regulated LINGO2, CD44, CD34, pVEGFR2, and N-Cadherin and down-regulated Occludin in LINGO2^high^ tumor tissues. (Arrows indicated).

We sorted SNU484 and N87 cells based on LINGO2 expression by flow cytometry (Figure 2A, Supplementary Figures S3A and S4A). The protein levels, motility, and tumorigenicity of SNU484 cells with high LINGO2 expression (LINGO2^high^) and lower LINGO2 expression (LINGO2^low^) were compared in vitro and in vivo. Higher expression of stemness-associated proteins including OCT4, PTEN, GLI1, and HEY1 was observed in LINGO2^high^ cells compared to LINGO2^low^. SNU484 LINGO2^high^ cells showed an approximately 2-fold increase (208% ± 24.6, *p* < 0.1) in cell migration and 4-fold increase (467% ± 15.8, *p* < 0.001) in clonogenic ability compared to SNU484 LINGO2^low^ cells (Figure 2B–D). N87 LINGO2^high^ cells also showed a similar increase in clonogenicity compared to the N87 LINGO2^low^ cells, in vitro (Supplementary Figure S3A). 

To determine tumor-initiating ability, sorted SNU484 cells were suspended in Matrigel and injected subcutaneously to the hind flanks of NOD/SCID mice (*n* = 3 per group). Tumor formation was observed with 250 LINGO2^high^ cells while LINGO2^low^ cells required more than 1000 cells to form a tumor mass (Figure 2E). Tumor mass formed from the same number of LINGO2^high^ and LINGO2^low^ cells differed in not only its size but also the overall color; LINGO2^high^ tumors were reddish whereas LINGO2^low^ tumors were nearly white. Similar results were observed when LINGO2^high^ and LINGO2^low^ cells were injected in BALB/c nude mouse (*n* = 1, Supplementary Figure S4B). We immuno-stained the mouse tissue slides for LINGO2, stemness marker CD44, angiogenesis marker phopho-vescular growth factor receptor 2 (p-VEGFR2), blood vessel marker CD34, mesenchymal marker N-Cadherin, and epithelial marker Occludin, followed by hematoxylin and eosin (H&E) staining (Figure 2F). SNU484 LINGO2^high^ tumors with up-regulated LINGO2 displayed up-regulated CD44, CD34, p-VEGFR2, and N-Cadherin but down-regulated Occludin compared to LINGO2^low^ tumors, suggesting the potential involvement of LINGO2 in angiogenesis and EMT. 

### 2.3. Silencing LINGO2 Reduces Cell Proliferation and Motility

**Figure 3:**
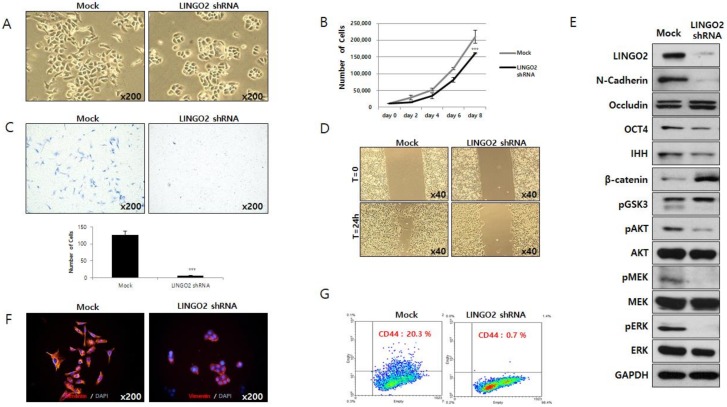
Silencing of LINGO2 reduces cell proliferation, cell motility, and cancer stem cell population. (**A**) Suppression of LINGO2 expression by shRNA changed the cell morphology from tapered ends to rounded ends. (**B**) Cell proliferation decreased by 23.6 ± 9.1% (*** *p* < 0.001) in LINGO2 shRNA cells. (**C**) Cell migration decreased by 95.5% ± 1.1 % (*** *p* < 0.001) in LINGO2 shRNA cells. (**D**) When cultures were scratched with a yellow tip, mock cells repaired the wounds in 24 h, but healing was delayed by approximately 6 hours in LINGO2 shRNA-transfected cells. (**E**) Proteins differentially expressed in LINGO2 shRNA-transfected cells were analyzed; stemness-associated proteins including OCT4, IHH, and β-Catenin; AKT/ERK signaling-related proteins including pGSK3, total AKT, phospho-AKT, phospho-MEK, total MEK, phospho-ERK, and total ERK; EMT-associated proteins including N-cadherin and Occludin. (**F**) Silencing LINGO2 decreased the expression of EMT marker, Vimentin visualized by immunofluorescent staining. (**G**) The CD44 population decreased from 20.3% to 0.7% by silencing LINGO2.

To determine the functional role of LINGO2, we suppressed LINGO2 expression in gastric cancer cell line SNU484 using shRNA. Cells transfected with LINGO2 shRNA became more rounded and cells with tapered ends disappeared (Figure 3A). LINGO2 silencing led to a decrease in SNU484 cell proliferation by 23.6% ± 9.1% (*p* < 0.001) and migration by 95.5% ± 1.1% (*p* < 0.001) (Figure 3B,C). Wound-healing ability was assessed, and wounds started to heal in 24 h in control cells while the healing process required more than 30 h in LINGO2 shRNA-transfected cells. Figure 3D shows the representative healing state at 24 h after creating the scratch in the cell monolayer.

We analyzed the effect of LINGO2 silencing on the expression of proteins (Figure 3E). When LINGO2 expression was suppressed stemness-associated proteins including OCT4, IHH, and β-Catenin expression was markedly altered—a decrease in OCT4 and IHH and an increase in β-Catenin was observed. Kinase B (AKT)/extracellular signal-regulated kinase (ERK) signaling-related proteins which are usually elevated in cancer cells were also altered with LINGO2 suppression—an increase in phospho-Glycogen synthase kinase-3 (pGSK3β) and a decrease in total AKT, phospho-AKT (pAKT), phospho-ERK kinase (pMEK), total ERK, and phospho-ERK (pERK) was observed. EMT-associated proteins including N-cadherin and Occludin were also altered in LINGO2 shRNA-transfected cells—a decrease in mesenchymal marker N-Cadherin and an increase in epithelial marker Occludin was observed. A marked decrease in mesenchymal marker Vimentin was also observed by immunofluorescence staining. A pronounced change in cell morphology was observed in LINGO2 shRNA-transfected cells (Figure 3F). Flow cytometry analyses revealed a decrease in stemness-associated cell surface marker CD44. The percentage of CD44-positive cells was 19.7 ± 0.85% and 0.8 ± 0.14% in mock and LINGO2 shRNA-transfected cells, respectively (Figure 3G).

### 2.4. LINGO2 Alters Secretion of Matrix Metallopeptidases and Angiogenic Factors

**Figure 4:**
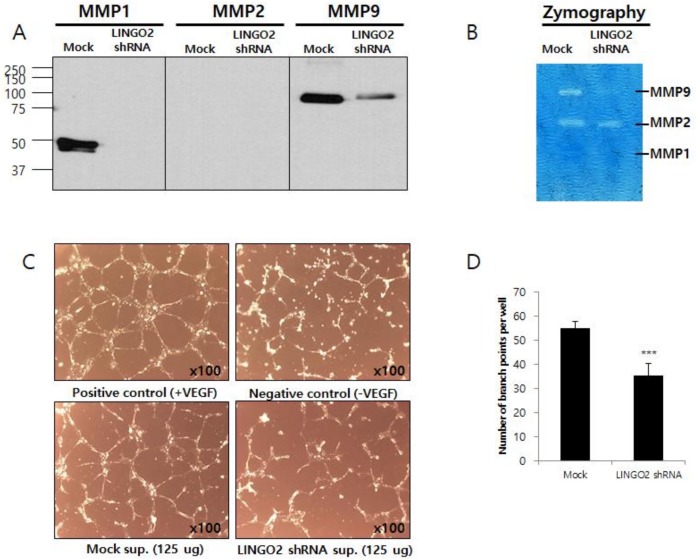
Silencing of LINGO2 reduces cell MMPs and human umbilical vein endothelial cells (HUVEC) tube formation (**A**) Reduced expression of MMP-1 and -9 proteins in the cultured supernatant and (**B**) active MMP-9 was observed in LINGO2 shRNA cells by western blot and zymography, respectively. (**C**,**D**) HUVEC tube formation was inhibited by LINGO2 shRNA culture supernatant by 35.6% ± 9.1% (*** *p* < 0.001).

We performed western blot analysis and gelatin zymography to assay the expression and activities of matrix metallopeptidases (MMPs). The activity of MMPs is critical for cancer cells to invade through extracellular matrices. Significant reduction in MMP-1 and -9 protein expression was observed in LINGO2-silenced cells whereas no MMP-2 was detected in either LINGO2-silenced or parent cells. Active MMP-9 levels were markedly reduced in LINGO2-silenced cells, whereas MMP-1 and MMP-2 expression levels remained unaltered on gelatin zymography (Figure 4A,B). Tube formation assay using human umbilical vein endothelial cells (HUVEC) in vitro assay to model the reorganization stage of angiogenesis was performed to measures the ability of endothelial cells to form capillary-like structures. Cell culture supernatants from LINGO2-silenced cells and mock translated cells supplemented with or without vesicular epithelial growth factor (VEGF) was used to culture HUVECs. LINGO2 silencing decreased tube formation by 35.6% ± 9.1% (*p* < 0.001) (Figure 4C,D).

### 2.5. Clinical Classification of Gastric Cancer Patients Based on LINGO2 Expression

**Figure 5:**
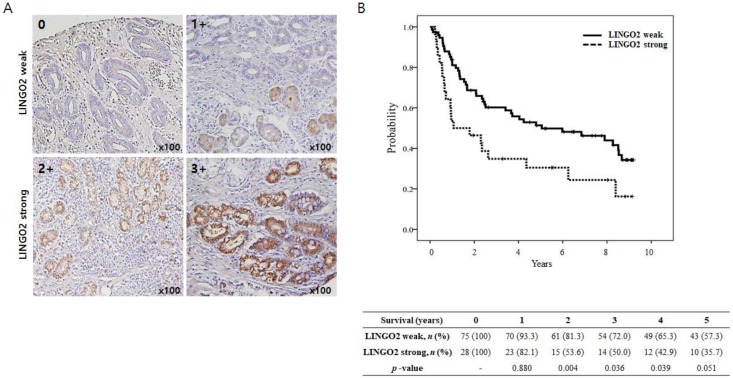
Kaplan-Meier estimates of overall survival (OS) according to LINGO2 expression. (**A**) LINGO2 expression in human gastric cancer tissues by immunohistochemistry. Tissue microarray samples of 103 gastric cancer tissues were stained with anti-LINGO2 antibody. LINGO2 staining was scored as a percentage of LINGO2-positive (+) tumor cells: no expression: 0, <20%: 1+, 20–50%: 2+, and >50%: 3+. 0 or 1+ was considered as LINGO2 weak whereas 2 or 3+ IHC staining scores of LINGO2 was considered LINGO2 strong group. (**B**) LINGO2 strong versus LINGO2 weak groups (*n*, 75 versus 28): median, O.S.; 121.2 versus 25.5 months; *p* = 0.012 by log-rank test. Yearly survival rate after surgery was significantly lower in LINGO2 strong group. (*p* = 0.004, 0.036 and 0.051 at 2, 3, and 5 years after surgery, respectively).

We evaluated the LINGO2 expression in 103 samples human gastric cancer by immunohistochemistry (IHC). Patients were divided into LINGO2 weak and LINGO2 strong groups based on LINGO2 expression. Patients with LINGO2 IHC staining scores of 0 or >1 were classified as LINGO2 weak group and patients with scores of >2 or >3 were classified as LINGO2 strong group (Figure 5A).

Table 1 presents the clinical characteristics of gastric cancer patients. Of the total 103 patients, 75 (72.8%) were classified as LINGO2 weak and 28 patients (27.2%) as LINGO2 strong. The study population consisted of 103 men (65.0%) with a mean age of 57.9 ± 10.7 years. The mean carcinoembryonic antigen (CEA) level at the time of diagnosis was 8.0 ng/mL. The overall survival (OS) was 96.9 months. Tumor, nodes, metastasis (TNM) stages were confirmed by pathology reports after surgery. Thirty-five patients (34.0%) were staged T1, 23 (22.3%) were T2, 43 (41.7%) were T3 and two patients (1.9%) were staged T4. Patients with N0, N1, N2, and N3 stage were 45 (43.7%), 27 (26.2%), 24 (23.3%), and 7 (6.8%), respectively. Distant metastasis was not observed in any patients.

The result of comparative analysis between LINGO2 weak and LINGO2 strong group are presented in Table 1. The LINGO2 strong group presented with a higher rate of advanced T stages (T3 or 4: 67.9% vs. 34.7%, *p* = 0.003), N stage (N2 or 3: 50.0% vs. 22.7%, *p* = 0.005) and an overall stage (stage III: 50.0% vs. 17.3%, *p* = 0.001). The OS was significantly shorter in LINGO2 strong group (25.5 vs. 121.2 months, *p* = 0.012) (Figure 5B). Moreover, the yearly survival rate after surgery was significantly lower in the LINGO2 strong group (*p* = 0.004, 0.036, 0.039, and 0.051 at 2, 3, 4, and 5 years after surgery, respectively).

Univariate analysis using Cox-regression identified that the LINGO2 strong group was significantly associated with OS (HR 1.939, *p* = 0.014), older age and advanced stage (all *p* < 0.001, Table 2). Subsequent multivariate analysis revealed that older age (*p* < 0.001) and advanced disease (*p* < 0.001) were significantly associated with OS. However, LINGO2 strong group was not associated with OS (HR 1.245, 95% CI 0.674–2.301, *p* = 0.484) after adjustment for the effect of other compounding factors by multivariate analysis using Cox-regression.

**Figure 6:**
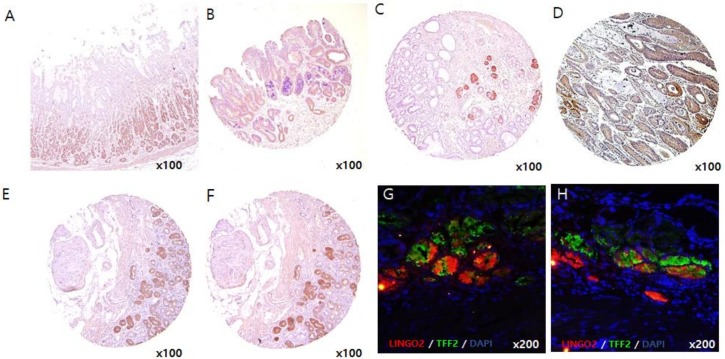
LINGO2 expression in (**A**) normal gastric tissue, (**B**) gastric polyp, (**C**) SPEM, and (**D**) gastric cancer tissues. Immunohistochemical staining of (**E**) LINGO2 expression and (**F**) TFF2, SPEM marker, with serially sectioned slides and (**G**,**H**) co-immunofluorescence staining of LINGO2 and TFF2 in separate patient tissue slides (LINGO2: red, TFF2: green, DAPI: blue).

Although LINGO2 gene was identified as a CSC marker from cancer cells and cancer sphere cells, it is possible that non-tumor cells also express LINGO2. We performed IHC and immunofluorescence staining on non-tumor gastric tissues, gastric polyp tissues and spasmolytic polypeptide-expressing metaplasia (SPEM) tissues along with gastric cancer tissues (Figure 6A–D). The expression of LINGO2 was barely detected in normal gastric tissues and gastric polyp tissues while moderate expression was observed in SPEM tissues. To visualize the location where LINGO2 expresses, we stained serial sections of SPEM tissues and stained with LINGO2 and SPEM marker, TFF2 (Figure 6E,F) [20,21,22,23,24]. Interestingly, cells expressing TFF2 also expressed LINGO2 which suggest that LINGO2 expresses in SPEM cells. To clarify the expression site, we performed immunofluorescence staining with both TFF2 and LINGO2 and co-expression was consistently observed in independent SPEM tissues (Figure 6G,H).

## 3. Discussion

LINGO2 is a novel protein encoded by the LINGO gene family first reported by Carim-Todd et al. in 2003; they observed LINGO2 expression in the central nervous system during early developmental stages and in the limbic system and cerebral cortex in adult tissues [25]. Haines et al. analyzed the *LINGO* expression during mouse embryonic development, reporting that *LINGO2* was expressed in neuronal tissues along with *LINGO4*, *NGL1*, *SALM1*, SALM5, and *TRKB* [26]. Vilarino-Guell et al. analyzed the expression of and mutations in *LINGO1* and *LINGO2*, reporting an association between *LINGO1* and *LINGO2* mutation and the risk of essential tremors and the incidence of Parkinson’s disease [18]. The association of LINGO2 with carcinogenesis has only been discussed with respect to gene location involved in different mutations. Klorin et al. reported that LINGO2 is one of the recurrent homozygous deleted genes in malignant mesothelioma cell line and revealed that the LINGO2 gene located in the proximal region of 9p21.2 was co-deleted with the 9p21.3 region of cyclin-dependent kinase inhibitor 2A (CDKN2A)—a well-known tumor suppressor [27]. Li et al. reported that genetic polymorphisms in the 9p21 region, which include the LINGO2 gene are associated with the risk of multiple cancers by single nucleotide polymorphism (SNP) analysis [28]. The potential role of the LINGO family in embryogenesis and in adult neuronal cells has been suspected; however, neither the mechanism of action of the LINGO family, including LINGO2, nor the potential molecular targets of any LINGO family member are yet known. 

The cancer stem cell theory, demonstrated by Reya et al. in 2001, proposes that cancers are derived from a stem cell compartment in a multistep process involving the accumulation of mutations in a variety of tumor suppressors and oncogenes [29]. CD44 is a well-known gastric CSC marker which is also a CSC marker in various other cancers [15,30]. Other markers including CD24/CD44, CD54/CD44, EpCAM/CD44, ALDH, CD90, and CD133 are also known to be gastric CSC markers. Cells expressing CSCs markers form cell-spheres and show increased cell motility [31,32,33,34,35,36,37]. The ability of a cell to form a sphere in nonadherent culture conditions is reflective of its self-renewal capacity. Cell-spheres are enriched in CSC population, and thus, can be used as sources to isolate CSCs [38,39]. We observed sphere-formation in two gastric cancer cell lines N87 and SNU484. The spheres passaged multiple times in vitro without the loss of sphere-forming capability or self-renewal capacity. Differentially expressed genes in the cell-spheres were analyzed by cDNA microarray analysis. Our studies identified LINGO2 as a novel CSC-associated gene.

In this study, we found that LINGO2 is expressed in gastric cancer tissues and regulates cell motility, tumorigenesis, and angiogenesis. Silencing LINGO2 by shRNA decreased stemness-associated genes including OCT4 and IHH as well as the CD44-positive cell population. LINGO2 silencing altered cell motility decreased N-cad, and Vimentin expression, as well as cell migration and wound-healing capacity. Cells highly expressing LINGO2 showed contrasting phenotypes including an elevated expression of stemness-associated molecules. LINGO2-silenced cells and LINGO2^low^ cells showed reduced in N-Cadherin, Vimentin, and MMPs, as well as cell migration compared to that of control vector, transfected cells, or LINGO2^high^ cells, suggesting that LINGO2 plays a role in EMT. LINGO2 silencing inhibited AKT and MEK/ERK phosphorylation, suggesting its role in the MEK-ERK signaling cascade. LINGO2^high^ showed an increase in tumorigenicity compared to LINGO2^low^ and generated a higher number of colonies in soft agar and required fewer cells to form a tumor in vivo compared to LINGO2^low^ cells. We performed tumorigenesis assay in two mouse strains NOD/SCID and BALBc/nude. LINGO2^high^ tumor mass showed a reddish color compared to that of white mass formed by LINGO2^low^ cells in both mouse models. This color difference suggested the possible relation of LINGO2 with angiogenesis. The elevated expression of pVEGFR2 and CD34 in LINGO2^high^ tumor tissues and the inhibition of HUVEC tube formation by cell culture supernatant from LINGO2-silenced cell supernatant which further supports the involvement of LINGO2 in angiogenesis. However, the specific role of LINGO2 in angiogenesis, cell motility, and stemness, and the mechanism underlying the LINGO2 mediated increase in stemness needs to be further investigated.

We analyzed LINGO2 expression in patient-derived tissues by IHC. We observed different levels of LINGO2 expression in all gastric cancer patients. Interestingly, patients with high LINGO2 expression (27%) showed an advanced clinical stage and decreased OS compared to patients with low LINGO2 expression (73%). These results were well supported by the functional analysis that silencing of LINGO2 reduced cell proliferation and motility in vitro, and tumor mass formation and angiogenesis were exaggerated in LINGO2^high^ cells than LINGO2^low^ cells in vivo. A more interesting finding was that LINGO2 expression was barely detected in both normal gastric tissues and adenomas polyp tissues, but a moderate expression, though not as strong as in cancer tissues, was observed in SPEM region in non-tumor gastric tissues. It is known that SPEM evolves from transdifferentiated chief cells triggered by the loss of parietal cells in the fundic mucosa [40]. With continuous inflammatory stimulation, intestinal metaplasia can occur from pre-existing SPEM and progress to cancer [41,42]. We observed the localization by immunofluorescence staining and LINGO2 expression was observed where TFF2, a known SPEM marker, was expressed. Therefore, LINGO2 was highly suspected to be associated with the development and growth of gastric cancer as well as the progression of gastric cancer from early to late stage. This is the first study to demonstrate the role of LINGO2 and its expression in human gastric cancer tissues.

## 4. Materials and Methods

### 4.1. Cell Culture

Human gastric cancer cell lines AGS, N87, SNU1, SNU5, and SNU16, and mouse fibroblast cell line NIH-3T3 were obtained from the American Type Culture Collection (ATCC, Manassas, VA, USA). Cell lines SNU484, SNU601, SNU638, and SNU668 were obtained from the Korea Cell Line Bank (KCLB, Seoul, Korea). All cells were maintained in RPMI1640 (Invitrogen Gibco, Grand Island, NY, USA) supplemented with 10% fetal bovine serum (FBS; Hyclone, Logan, UT, USA), at 37 °C in a humidified incubator with 5% CO_2_.

### 4.2. Sphere-Formation Assay

Cells were trypsinized and resuspended at a density of 1.0 × 10^3^ cells/well in D/F12 (Invitrogen Gibco) medium supplemented with 10 ng/mL epidermal growth factor (R&D Systems Inc., Minneapolis, MN, USA), 10 ng/mL basic fibroblast growth factor (R&D Systems Inc.), 1× insulin-transferring selenium (Invitrogen, Carlsbad, CA, USA), 0.5% bovine serum albumin (Invitrogen), and 0.5% FBS in ultra-low attachment culture plates (Corning Inc., Corning, NY, USA). Adherent cultured cells, as a control, were seeded in culture dishes (Nalgene Nunc Intl, Rochester, NY, USA) with sphere-formation medium. After seven days, cells were collected and dissociated with Accutase (Sigma-Aldrich, St. Louis, MO, USA).

### 4.3. Microarray Analysis

Total RNA was extracted from adherent cells and cell-spheres from N87 and SNU484 gastric cancer cell lines using RNeasy Mini kits (Qiagen, Valencia, CA, USA). Microarray procedures were carried out according to the manufacture0072’s protocols. Briefly, 6-μg aliquots of total RNA were used to prepare double-stranded cDNA. cDNAs were amplified by PCR and labelled with biotin using the IVT labelling kit (Affymetrix, Santa Clara, CA, USA). Labelled cRNA was fragmented and hybridized to an Affymetrix GeneChip Human Genome U133 plus 2.0 high-density oligonucleotide arrays (Affymetrix). Microarrays were then washed using a GeneChip Fluidics Station 450 (Affymetrix) and scanned using a GeneChip Array Scanner 3000 7G (Affymetrix). Expression data were generated using Affymetrix Expression Console software version 1.1 using MAS5 algorithm normalization. Expression intensity data in CEL file was normalized using the MAS5 algorithm to reduce noise.

### 4.4. Knockdown of LINGO2 by shRNA

To verify the effects of LINGO2 inhibition, LINGO2 shRNA plasmid or mock shRNA plasmid (Sure Silencing shRNA plasmids, Qiagen) was transfected into SNU484 cells using Lipofectamine 2000 (Invitrogen) followed by treatment with puromycin (2 μg/mL) to establish a LINGO2 knockdown cell line. The LINGO2 shRNA sequence was 5′-AGACTTGAGTGACAACATCAT-3′ and the mock shRNA was 5′-GGAATCTCATTCGATGCATAC-3′. 

### 4.5. Semiquantitative Reverse-Transcription Polymerase Chain Reaction (RT-PCR)

Total RNA was extracted for RT-PCR using RNeasy Mini kits (Qiagen), and complementary single-strand DNA was synthesized using a Superscript II system (Invitrogen) according to the manufacturers’ protocols. *LINGO2* (forward primer 5′-TTGCAAATATTGGCGTTCTG-3′; reverse primer 5′-TGATGCAAGGCTTTAACAAATG-3′) expression was evaluated in gastric cancer spheres and control cells. *β-Actin* (forward primer 5′-GGCATCCTCACCCTGAAGTA-3′; reverse primer 5′-GGGGTGTTGAAGGTCTCAAA-3′) was used as a reference gene. Primer sequences are supplied in Supplementary Table S2.

### 4.6. Western Blotting

Cells were lysed in a buffer composed of 70 mM glycerophosphate (pH 7.2), 0.6 mM Na vanadate, 2 mM MgCl2, 1 mM EGTA, 1 mM dithiothreitol, 0.5% Triton X-100, 0.2 mM phenylmethylsulfonyl fluoride, and 1× complete protease inhibitor (Roche Applied Science, Nutley, NJ, USA). Proteins (25 μg) were separated on sodium dodecyl sulphate-polyacrylamide gels and transferred to a polyvinylidene difluoride membrane (Immobilon-P; Millipore, Bedford, MA, USA) that was blocked in 5% (w/v) non-fat dry milk (Bio-Rad Laboratories, Hercules, CA, USA) and incubated overnight at 4°C with antibodies diluted 1:1000. Primary antibodies to mouse monoclonal anti-LINGO2 (from LifeSpan BioSciences Inc., Seattle, WA, USA); mouse monoclonal anti-N-Cadherin, mouse monoclonal anti-MMP2, rabbit monoclonal anti-MMP9 (from Abcam, Cambridge, UK); rabbit polyclonal anti-OCT4, rabbit polyclonal anti-phospho-GSK3, rabbit polyclonal anti-phospho-kinase B (pAKT), rabbit polyclonal anti-phospho-MEK, and rabbit polyclonal anti-phospho-ERK (from Cell Signaling Technology, Inc., Danvers, MA, USA); rabbit polyclonal anti-Occludin, mouse monoclonal anti-PTEN, rabbit polyclonal anti-GLI1, rabbit polyclonal anti-HEY1, rabbit polyclonal anti-IHH, mouse monoclonal anti-β-Catenin, rabbit polyclonal anti-AKT, rabbit polyclonal anti-MEK, rabbit polyclonal anti-ERK, mouse monoclonal anti-MMP1, and mouse monoclonal anti-GAPDH (all from Santa Cruz Biotechnology, CA, USA) were used. Horseradish peroxidase-conjugated goat anti-mouse IgG and goat anti-rabbit IgG secondary antibodies (both from Santa Cruz Biotechnology) were used for detection and immunoblots were developed with Super Signal^®^ West Pico Chemiluminescent substrate (Thermo Scientific, Rockford, IL, USA). 

### 4.7. Immunofluorescence Staining

Cultured cells were fixed in methanol and immunostained with mouse monoclonal anti-Vimentin (1:100, Santa Cruz Biotechnology) overnight at 4 °C. Stained cells were incubated with goat anti-mouse Cy3-conjugated secondary antibody (Jackson ImmunoResearch Inc., West Grove, PA, USA) for 30 min. Nuclei were labelled with 4′,6-diamidino-2-phenylindole (DAPI). Stained cells were analyzed on an Olympus BX51 microscope and images captured using an Olympus DP71 camera (Olympus America Inc., Center Valley, PA, USA). Tissue slides were were deparaffinized in xylene and rehydrated in graded alcohol. Endogenous peroxidase activity was blocked with 0.3% (*v*/*v*) hydrogen peroxide in methanol. Antigen retrieval was performed by microwaving the slides in sodium citrate buffer (0.01 M, pH 6.0) for 5 min. To block nonspecific staining, sections were incubated with 10% (*v*/*v*) normal donkey serum for 1 h and incubated with mouse monoclonal anti-LINGO2 (1:50, LifeSpan BioSciences Inc.) and mouse monoclonal anti-TFF2 (1:25, Leica Biosystems Newcastle Ltd., Newcastle Upon Tyne, UK) overnight at 4 °C. The slides were visualized using Cy3-goat anti-mouse IgG_2a_ (LINGO2, 1:100, Jackson ImmunoResearch Inc.) and Alexa488-Donkey anti-mouse IgM (TFF2, 1:50, Jackson ImmunoResearch Inc.) in antibody diluent. and incubated for 30min at room temperature. Between each step, there were three washing steps of 5min each on a rocking platform in PBS. The slides were cover slipped using mounting medium for fluorescence with DAPI (Vecta shield H-1200; Vector Laboratories, Inc. Burlingame, CA, USA).

### 4.8. Cell Proliferation Assays

Cells were detached and plated in triplicate at a density of 1.0 × 10^4^ cells/well in 24-well plates in complete medium. Cells were detached every two days for eight days and stained with Trypan blue (Sigma-Aldrich, St. Louis, MO, USA) and counted using a hematocytometer.

### 4.9. Migration and Invasion Assays

For the migration assay, cells were detached and suspended at 1.0 × 10^5^ cells/mL in serum-free media and plated at a density of 1.0 × 10^4^ cells/well in 24-well Transwell plates (Costar, Bethesda, MD, USA). For the invasion assay, the upper chamber was pre-coated with Matrigel (1:4 diluted with serum-free medium, BD Biosciences, San Jose, CA, USA), and cells were seeded at 1.0 × 10^4^ cells/well. The bottom chamber was filled with the culture medium from NIH-3T3 fibroblasts. Cells were incubated for 24 h for migration assays and 72 h for invasion assays. After incubation, cells were fixed with 5% glutaraldehyde for 30 min and stained with 0.1% crystal violet. Cells were completely removed from the upper surface of the membrane with a moist cotton swab. Migrated and invaded cells were counted and photographed under a microscope at 100× magnification. All assays were performed in triplicate.

### 4.10. Wound-Healing Assays

A scratch was made across a cell culture dish at approximately 90–95% cell confluence using a yellow pipet tip. The cells were incubated at 37 °C and photographed at 40× magnification during the time course of healing.

### 4.11. Clonogeinc Assays

For colony formation, the culture plate was pre-coated with base agar 0.6%, and cells were resuspended with 500 cells/well in top agar 0.3% with complete medium in 24-well plates. The plates were incubated at 37 °C with 5% CO_2_ until colonies were visible. The colonies were then stained with 0.01% crystal violet and counted under an inverted microscope.

### 4.12. Gel Zymography

Cells were incubated in serum-free medium for 48 h, and the culture medium was collected and concentrated using Amicon ultrafilter devices (Millipore, Tullagreen, Ireland). Samples were loaded with non-reducing loading dye and electrophoresed through 10% zymogram gels (Bio-rad, Hercules, CA, USA). SDS was removed from the gels by incubation in 2.5% Triton X-100 (Sigma), and the gels were incubated at 37 °C for 16 h in development buffer (50 mM Tris-HCl, pH7.8, 200 mM NaCl, 5 nM CaCl_2_, 0.02% Brij-35), followed by staining with Coomassie blue. MMP activity was visualized as clear bands.

### 4.13. Flow Cytometry

Cells were dissociated with Accutase (Sigma-Aldrich) and resuspended in Hank’s balanced salt solution (HBSS) with 2% FBS at 10^6^ per 100 μL. Cells were stained with anti-LINGO2 (Lifespan Biosciences, Inc.) or purified mouse IgG2a, κ isotype (BD Biosciences PharMingen, San Diego, CA, USA) as a control for 20 min on ice and washed twice with HBSS/2% FBS. Secondary antibody phycoerythrin goat anti-mouse IgG (BD Biosciences PharMingen), was added to cells resuspended in HBSS/2% FBS followed by a 20 min incubation on ice. FACSAria II (BD Immunocytochemistry System, Franklin Lakes, NJ, USA) was used for flow cytometry. To analyze CD44 expression, cells were dissociated with Accutase (Sigma-Aldrich) and 1 × 10^6^ cells were labelled with anti-CD44-fluorescein isothiocyanate (BD Biosciences PharMingen). Labelled cells were detected using a BD LSRII flow cytometer (BD Biosciences, Franklin Lakes, NJ, USA).

### 4.14. Tumorigenesis Assays

Gastric cancer cells sorted as LINGO2^high^ and LINGO2^low^ by flow cytometry, were washed with serum-free HBSS (Gibco BRL, Grand Island, NY, USA) and suspended in serum-free RPMI (Gibco BRL) and Matrigel (BD Biosciences PharMingen) (1:1 volume). Cells were subcutaneously injected into both flanks of NOD/SCID mice (*n* = 3 per group, Charles River Laboratories, Yokohama, Japan). The animal experiments were approved by the Committee for the Care and Use of Laboratory Animals of Yonsei University College of Medicine. Tumor volume was calculated as V (mm^3^) = (A^2^ × B)/2, where A is the diameter perpendicular to the largest dimension B. After 14 to 16 weeks, mice were sacrificed and tumor tissues fixed in 4% paraformaldehyde. For histological evaluation, tissue samples were embedded in paraffin and stained with hematoxylin and eosin (H & E, Sigma-Aldrich).

### 4.15. Tube Formation Assay

Matrigel (BD Biosciences) was diluted with EBM-2 medium and coated in 96-well plates at 37 °C for 1 h. Then, 4 × 10^3^ human umbilical vein endothelial cells (HUVECs) were seeded in the EBM-2 medium on Matrigel. The tube formation ability of HUVECs was measured at 6 h with RPMI with or without VEGF, or concentrated cell culture media. After incubation, the number of tubes and nodes of the tubular structures was quantified.

### 4.16. Patients

We performed a retrospective analysis of surgical tissue samples of gastric cancer patients between January 2002 and December 2002. All patients included in the study received gastric cancer surgery with curative intention. Information regarding patient demographics and clinical data was obtained from the electronic medical records, including age at diagnosis, sex, tumor stages at diagnosis, and serum levels of CEA. Tumors were staged based on the staging classification of the 7th edition of the American Joint Committee on Cancer (AJCC). All procedures involving human participants were performed in accordance with the ethical standards of the institutional research committee and the 1964 Helsinki declaration and its later amendments or comparable ethical standards. As this was a retrospective analysis, Yonsei University Health System, Severance Hospital, Institutional Review Board approval was obtained and the need for informed consent was waived-off.

### 4.17. Immunohistochemistry

A total of 103 human gastric cancer tissues from surgical resections at Severance Hospital, Yonsei University College of Medicine were used. Gastric cancer tissue samples were embedded in paraffin and stained with H & E. After pathological evaluation, tumor tissues were arrayed into new paraffin blocks for tissue microarrays (TMAs). The Ethical Committee for the Clinical Research of the Institutional Review Board of Severance Hospital, Yonsei University College of Medicine, Seoul, Korea, approved this study protocol (IRB approval code 4-2007-0261, 30 August 2007). TMA sections were deparaffinized in xylene and rehydrated in graded alcohol. Endogenous peroxidase activity was blocked with 0.3% (*v*/*v*) hydrogen peroxide in methanol. Antigen retrieval was performed by microwaving the slides in sodium citrate buffer (0.01 M, pH 6.0) for 5 min. To block nonspecific staining, sections were incubated with 10% (*v*/*v*) normal donkey serum for 1 h and incubated with mouse monoclonal anti-LINGO2 (1:500, LifeSpan BioSciences, Inc.) and mouse monoclonal anti-TFF2 (1:50, Leica Biosystems Newcastle Ltd.) overnight at 4 °C. Subsequent reactions were performed using Envision kits (DakoCytomation California, Inc., Carpinteria, CA, USA) following the manufacturer’s instructions. Immunoreactions were developed with DAKO Liquid DAB+ substrate-chromogen system and counterstained with Harris hematoxylin (Sigma-Aldrich). Immunoreactivity was scored as percentage of LINGO2-positive tumor cells– no expression: 0, <20%: 1+, 20-50%: 2+, and >50%: 3+. Mouse tissue slides were incubated overnight at 4°C with mouse monoclonal anti-LINGO2 (1:200, LifeSpan BioSciences, Inc.), rabbit polyclonal anti-pVEGFR2 (1:50, Cell Signaling Technology), rabbit polyclonal anti-Occludin (1:200, Santa Cruz Biotechnology), mouse monoclonal anti-CD44 antibody (1:100, Neomarkers Inc., Fremont, CA, USA), mouse monoclonal anti-N-Cadherin antibody (1:100, Abcam), and rat monoclonal anti-mouse CD34 antibody (1:200, Abcam) in antibody diluent. The reaction was subsequently carried out with an LSAB+ Kit (Dako), and sections were counterstained with Mayer’s hematoxylin, dehydrated, and observed under a BX51 microscope (Olympus, Tokyo, Japan).

### 4.18. Statistical Analysis

Data were analyzed using χ^2^ and Fisher’s exact for categorical data and Student’s t-test and Mann-Whitney U test for continuous variables. Multivariate analysis was performed to evaluate possible significant factors, considering the influence of confounding clinical variables. Hazard ratios (HRs), 95% confidence intervals (95% CIs), and P values of multivariate analysis were calculated with a Cox proportional hazard model for OS. OS was estimated and compared using the Kaplan-Meier analysis with a log-rank test. All statistical analyses were performed using IBM SPSS Statistics for Windows, version 23.0 (IBM Corp., Armonk, NY, USA). A value of *p* < 0.05 was considered statistically significant. Asterisks (*, **, and ***) indicate significances at *p* ≤ 0.05, *p* < 0.1, and *p* < 0.01, respectively.

## 5. Conclusions

Our study reports for the first time that LINGO2 expression is elevated in stemness-enriched cells in gastric cancer cell lines, based on cDNA microarray analysis, and evaluation of tissues derived from gastric cancer patients. Furthermore, novel functions of LINGO2 in regulating cell migration, stemness, MMPs, tumorigenic ability, and angiogenesis were demonstrated, as well as the first clinical implication of LINGO2 expression correlated with gastric cancer progression. We propose that therapeutic targeting of LINGO2 may contribute to target gastric CSC to overcome conventional cancer therapy.

## Figures and Tables

**Table 1:** Clinical characteristics based on LINGO2 expression.

	Total *n* = 103	LINGO2 Weak *n* = 75	LINGO2 Strong *n* = 28	*p*
Mean age, years (SD)	57.9 (10.7)	58.3 (10.5)	56.9 (11.3)	0.549
Sex (%)				
Male	67 (65.0)	50 (66.7)	17 (60.7)	0.573
Female	36 (35.0)	25 (33.3)	11 (39.3)	
CEA, ng/mL (SD)	8.0 (26.3)	8.0 (26.3)	8.1 (27.1)	0.992
Median OS, months (range)	96.9 (2.0–220.8)	121.2 (2.0–220.8)	25.5 (5.3–219.3)	0.012
pT stage (%)				0.011
T1	35 (34.0)	29 (38.7)	6 (21.4)	
T2	23 (22.3)	20 (26.7)	3 (10.7)	
T3	43 (41.7)	24 (32.0)	19 (67.9)	
T4	2 (1.9)	2 (2.7)	0 (0.0)	
T1/2	58 (56.3)	49 (65.3)	9 (32.1)	0.003
T3/4	45 (43.7)	26 (34.7)	19 (67.9)	
pN stage (%)				0.005
N0	45 (43.7)	39 (52.0)	6 (21.4)	
N1	27 (26.2)	19 (25.3)	8 (28.6)	
N2	24 (23.3)	15 (20.0)	9 (32.1)	
N3	7 (6.8)	2 (2.7)	5 (17.9)	
N0/1	72 (69.9)	58 (77.3)	14 (50.0)	0.007
N2/3	31 (30.1)	17 (22.7)	14 (50.0)	
Stage (%)				0.015
IA	26 (25.2)	23 (30.7)	3 (10.7)	
IB	28 (27.2)	22 (29.3)	6 (21.4)	
IIB	22 (21.4)	17 (22.7)	5 (17.9)	
IIIA	18 (17.5)	9 (12.0)	9 (32.1)	
IIIB	9 (8.7)	4 (5.3)	5 (17.9)	
I/II	76 (73.8)	62 (82.7)	14 (50.0)	0.001
III	27 (26.2)	13 (17.3)	14 (50.0)	

**Table 2:** Cox proportional hazard analysis for the contribution of clinical factors to OS.

	Univariate		Multivariate	
	Hazard Ratio (95% CI)	*p*	Hazard Ratio (95% CI)	*p*
LINGO2strong (vs. LINGO2 weak)	1.939 (1.146–3.279)	0.014	1.245 (0.674–2.301)	0.484
Older Age	1.053 (1.025–1.082)	<0.001	1.053 (1.023–1.083)	<0.001
Male sex (vs. female)	1.160 (0.689–1.956)	0.576	1.433 (0.805–2.551)	0.222
Higher initial CEA	1.007 (0.998–1.016)	0.141	1.001 (0.991–1.011)	0.828
Stage (III vs. I or II)	3.768 (2.235–6.354)	<0.001	3.693 (2.059–6.624)	<0.001

## Supplementary Materials

Supplementary materials can be found at http://www.mdpi.com/1422-0067/20/3/555/s1.

## Abbreviations

LINGO2	leucine-rich repeat and immunoglobulin-like domain-containing nogo receptor-interacting protein 2
CSC	cancer stem cells
EMT	epithelial to mesenchymal transition
TMA	tissue microarray analysis
SPEM	spasmolytic polypeptide-expressing metaplasia
