# Supplementary material for: Novel Gastric Cancer Stem Cell-Related Marker LINGO2 Is Associated with Cancer Cell Phenotype and Patient Outcome
Source: Int J Mol Sci. 2019 Jan 28;20(3):555. doi: 10.3390/ijms20030555 (PMC6387145; doi:10.3390/ijms20030555)
Supplement: Supplementary file 1 [file ijms-20-00555-s001.zip › supplementary/Supplementary Tables.pdf]

Supplementary Table 1. Differentially expressed genes in SNU484 and N87 sphere (Fold change=sphere/adherent)

	Fold Change (sphere/adh)				Fold Change (sphere/adh)		
Gene symbol	SNU484	N87	Gene name	Gene symbol	SNU484	N87	Gene name
EDN3	8.36	1.33	endothelin 3	RAB31	-2.20	-9.21	RAB31, member RAS oncogene family
BAI3	6.11	1.96	brain-specific angiogenesis inhibitor 3	NAV2	-2.21	-2.23	neuron navigator 2
LRRN3	5.64	1.68	leucine rich repeat neuronal 3	CAV1	-2.22	-3.80	caveolin 1, caveolae protein, 22kDa
CALB1	4.53	1.23	calbindin 1, 28kDa	CD24	-2.22	-9.20	CD24 molecule
KLHL1	4.49	1.22	kelch-like 1 (Drosophila)	DDIT4	-2.23	-1.30	DNA-damage-inducible transcript 4
LMO3	4.37	1.50	LIM domain only 3 (rhombotin-like 2)	SCIN	-2.23	-1.63	scinderin
POSTN	4.26	1.25	periostin, osteoblast specific factor	FH	-2.24	-1.47	fumarate hydratase
CHGB	4.19	1.44	chromogranin B (secretogranin 1)	IGFBP6	-2.27	-1.82	insulin-like growth factor binding protein 6
FAT4	3.93	1.44	FAT tumor suppressor homolog 4 (Drosophila)	EGFR	-2.28	-1.91	epidermal growth factor receptor
CDH19	3.68	1.78	cadherin 19, type 2	PROCR	-2.28	-2.18	protein C receptor, endothelial (EPCR)
ADAM22	3.60	1.32	ADAM metallopeptidase domain 22	GRK6	-2.30	-1.39	G protein-coupled receptor kinase 6
DSC1	3.43	1.28	desmocollin 1	SH3BP4	-2.32	-1.45	SH3-domain binding protein 4
CSMD3	3.22	1.39	CUB and Sushi multiple domains 3	SOX9	-2.33	-5.96	SRY (sex determining region Y)-box 9
GRAMD3	3.08	1.65	GRAM domain containing 3	CBX5	-2.36	-2.40	Chromobox homolog 5 (HP1 alpha homolog, Drosophila)
CNTNAP3	2.98	1.27	contactin associated protein-like 3 /// contactin associated protein-like 3B	AKAP12	-2.36	-2.50	A kinase (PRKA) anchor protein (gravin) 12
**LINGO2**	2.95	1.37	Leucine rich repeat and Ig domain containing 2	NNMT	-2.38	-5.47	nicotinamide N-methyltransferase
MUC15	2.94	1.21	mucin 15, cell surface associated	PAPPA	-2.38	-4.42	pregnancy-associated plasma protein A, pappalysin 1
SYCP2	2.93	1.69	synaptonemal complex protein 2	SGK1	-2.39	-1.93	serum/glucocorticoid regulated kinase 1
IGF1	2.92	1.36	insulin-like growth factor 1 (somatomedin C)	COL1A1	-2.39	-3.61	collagen, type I, alpha 1
HGF	2.85	1.27	hepatocyte growth factor (hepapoietin A; scatter factor)	MDFIC	-2.41	-1.69	MyoD family inhibitor domain containing
LRFN5	2.81	2.48	leucine rich repeat and fibronectin type III domain containing 5	SOCS3	-2.45	-1.72	suppressor of cytokine signaling 3
NEBL	2.77	1.32	Nebulette	NAV3	-2.47	-2.22	neuron navigator 3
FOLR1	2.76	1.20	folate receptor 1 (adult)	DTNA	-2.47	-1.27	dystrobrevin, alpha
SCUBE3	2.69	1.25	signal peptide, CUB domain, EGF-like 3	FSTL3	-2.47	-1.34	follistatin-like 3 (secreted glycoprotein)
PLXDC2	2.66	1.30	plexin domain containing 2	PSAT1	-2.48	-1.33	phosphoserine aminotransferase 1
11-3	2.62	1.32	membrane-associated ring finger (C3HC4) 11	CD36	-2.50	-3.78	CD36 molecule (thrombospondin receptor)
LIPA	2.61	1.21	lipase A, lysosomal acid, cholesterol esterase (Wolman disease)	GIGYF2	-2.51	-2.11	trinucleotide repeat containing 15
PCDH9	2.61	1.28	protocadherin 9	C10orf47	-2.52	-1.70	chromosome 10 open reading frame 47
NLGN1	2.55	1.22	neuroligin 1	SPHK1	-2.54	-1.72	sphingosine kinase 1
CEP350	2.54	1.29	centrosomal protein 350kDa	2orf59 /// LOC54147	-2.57	-1.29	chromosome 2 open reading frame 59 /// hypothetical LOC541471
LONP2	2.51	2.34	Seven in absentia homolog 1 (Drosophila)	SERPINB2	-2.58	-16.28	serpin peptidase inhibitor, clade B (ovalbumin), member 2
MALAT1	2.51	1.44	PRO1073 protein	EFEMP1	-2.61	-8.65	EGF-containing fibulin-like extracellular matrix protein 1
BCL11A	2.48	1.60	B-cell CLL/lymphoma 11A (zinc finger protein)	FAM84B	-2.63	-2.96	family with sequence similarity 84, member B
ID4	2.47	2.45	Inhibitor of DNA binding 4, dominant negative helix-loop-helix protein	PHLDA1	-2.65	-1.26	pleckstrin homology-like domain, family A, member 1
JAM2	2.46	1.21	junctional adhesion molecule 2	PSMB9	-2.67	-8.96	proteasome (prosome, macropain) subunit, beta type, 9
ID2 /// ID2B	2.43	6.49	inhibitor of DNA binding 2, dominant negative helix-loop-helix protein	C12orf39	-2.68	-1.37	chromosome 12 open reading frame 39
KIAA2022	2.38	1.33	KIAA2022	GADD45B	-2.72	-1.25	growth arrest and DNA-damage-inducible, beta
ANO3	2.38	1.41	transmembrane protein 16C	SEPP1	-2.73	-2.67	selenoprotein P, plasma, 1
GPATCH2	2.37	2.52	G patch domain containing 2	ITGA2	-2.74	-2.46	integrin, alpha 2 (CD49B, alpha 2 subunit of VLA-2 receptor)
100134366 /// PLGLA	2.34	3.20	plasminogen-like B2 /// plasminogen-like B1 /// plasminogen-like A1	CLU	-2.77	-1.51	clusterin
DPY19L2	2.32	1.72	dpy-19-like 2 (C. elegans)	DKK3	-2.82	-7.11	dickkopf homolog 3 (Xenopus laevis)
SUV420H1	2.32	1.31	suppressor of variegation 4-20 homolog 1 (Drosophila)	PRSS23	-2.83	-1.82	protease, serine, 23
MSL-1	2.30	1.42	male-specific lethal-1 homolog	ABCF2	-2.88	-1.60	ATP-binding cassette, sub-family F (GCN20), member 2
SDC2	2.24	1.86	syndecan 2	EMP1	-2.91	-1.25	epithelial membrane protein 1
HOMER1	2.23	1.62	homer homolog 1 (Drosophila)	QKI	-2.92	-1.21	quaking homolog, KH domain RNA binding (mouse)
P2RY1	2.21	1.39	purinergic receptor P2Y, G-protein coupled, 1	PALLD	-2.94	-1.86	palladin, cytoskeletal associated protein
TRIM73	2.21	1.32	tripartite motif-containing 73	MICB	-2.99	-2.17	MHC class I polypeptide-related sequence B
PLAG1	2.21	1.81	pleiomorphic adenoma gene 1	CD55	-3.04	-2.42	CD55 molecule
DHX9	2.19	1.75	DEAH (Asp-Glu-Ala-His) box polypeptide 9	MICAL2	-3.05	-1.85	microtubule associated monoxygenase, calponin and LIM domain containing 2
FZD7	2.16	1.37	frizzled homolog 7 (Drosophila)	PTPRK	-3.06	-1.75	protein tyrosine phosphatase, receptor type, K
TTC18	2.16	1.33	tetratricopeptide repeat domain 18	BIRC3	-3.08	-1.98	baculoviral IAP repeat-containing 3
KDR	2.16	1.36	kinase insert domain receptor (a type III receptor tyrosine kinase)	SLC16A6	-3.11	-1.24	solute carrier family 16, member 6 (monocarboxylic acid transporter 7)
EPHA5	2.15	1.36	EPH receptor A5	DST	-3.15	-1.37	dystonin
ZMAT1	2.15	1.31	zinc finger, matrin type 1	PLAU	-3.18	-2.67	plasminogen activator, urokinase
C5orf13	2.15	1.21	chromosome 5 open reading frame 13	TM4SF1	-3.19	-2.19	transmembrane 4 L six family member 1
COL21A1	2.12	1.30	collagen, type XXI, alpha 1	CYR61	-3.24	-1.38	cysteine-rich, angiogenic inducer, 61
NUCB2	2.11	1.21	nucleobindin 2	TFAP2C	-3.29	-1.79	transcription factor AP-2 gamma
ZNF436	2.11	1.79	zinc finger protein 436	ULBP2	-3.31	-2.48	UL16 binding protein 2
ARSD	2.10	1.26	arylsulfatase D	SPOCK1	-3.34	-2.14	sparc/osteonectin, cwcv and kazal-like domains proteoglycan (testican) 1
WDR52	2.04	1.29	WD repeat domain 52	CAV2	-3.37	-2.73	caveolin 2
AMH	2.04	3.13	anti-Mullerian hormone	COTL1	-3.42	-2.29	coactosin-like 1 (Dictyostelium)
CPE	2.03	1.47	carboxypeptidase E	AADACL1	-3.60	-1.60	arylacetamide deacetylase-like 1
C3orf21	2.02	1.28	chromosome 3 open reading frame 21	TOX2	-3.65	-3.25	TOX high mobility group box family member 2
SGSH	2.02	1.30	N-sulfoglucosamine sulfohydrolase (sulfamidase)	SAMD9	-3.76	-1.79	sterile alpha motif domain containing 9
MZF1	2.01	1.38	myeloid zinc finger 1	FHL2	-3.85	-2.55	four and a half LIM domains 2
ARMCX3	2.01	1.37	armadillo repeat containing, X-linked 3	LOX	-3.93	-1.22	lysyl oxidase
ASNS	-2.00	-1.25	asparagine synthetase	LAMP3	-4.05	-1.81	lysosomal-associated membrane protein 3
AHNAK2	-2.01	-1.86	AHNAK nucleoprotein 2	OBFC2A	-4.25	-1.28	oligonucleotide/oligosaccharide-binding fold containing 2A
PTK2	-2.02	-2.79	PTK2 protein tyrosine kinase 2	TNS3	-4.38	-1.66	tensin 3
CRYZL1	-2.02	-1.57	crystallin, zeta (quinone reductase)-like 1	ARHGAP29	-4.49	-5.28	Rho GTPase activating protein 29
CCND1	-2.03	-1.38	cyclin D1	NOX4	-4.58	-1.33	NADPH oxidase 4
LY6E	-2.04	-1.23	lymphocyte antigen 6 complex, locus E	DOCK10	-4.65	-1.70	dedicator of cytokinesis 10
CCL5	-2.04	-2.41	chemokine (C-C motif) ligand 5	THBS1	-4.66	-1.54	thrombospondin 1
F2R	-2.05	-6.84	coagulation factor II (thrombin) receptor	SLC6A15	-4.88	-4.71	solute carrier family 6, member 15
FOXF1	-2.06	-5.27	forkhead box F1	PHLDA2	-4.98	-1.54	pleckstrin homology-like domain, family A, member 2
SNCA	-2.07	-1.68	synuclein, alpha (non A4 component of amyloid precursor)	NT5E	-5.21	-7.42	5'-nucleotidase, ecto (CD73)
CLIC3	-2.08	-1.56	chloride intracellular channel 3	MYO10	-5.36	-1.84	myosin X
TRIP10	-2.08	-1.26	thyroid hormone receptor interactor 10	ARL4C	-5.82	-1.21	ADP-ribosylation factor-like 4C
FGF2	-2.10	-1.31	fibroblast growth factor 2 (basic)	CXCL1	-5.89	-1.84	chemokine (C-X-C motif) ligand 1 (melanoma growth stimulating activity, alpha)
CLDN1	-2.10	-1.90	Claudin 1	F2RL1	-6.61	-11.89	coagulation factor II (thrombin) receptor-like 1
PRPS1	-2.10	-1.50	phosphoribosyl pyrophosphate synthetase 1	CALD1	-6.61	-1.33	caldesmon 1
kazrin	-2.11	-1.54	kazrin	RBM24	-6.82	-2.92	RNA binding motif protein 24
KCNN4	-2.12	-2.92	potassium intermediate/small conductance calcium-activated channel, subfamily N, member 4	GEM	-7.68	-1.81	GTP binding protein overexpressed in skeletal muscle
CDKN2AIPNL	-2.13	-1.63	CDKN2A interacting protein N-terminal like	DNER	-10.62	-2.52	delta/notch-like EGF repeat containing
PDLIM7	-2.13	-1.38	PDZ and LIM domain 7 (enigma)	SRPX	-13.04	-4.94	sushi-repeat-containing protein, X-linked
SWAP70	-2.13	-1.59	SWAP-70 protein	IL8	-16.30	-2.26	interleukin 8
KLF5	-2.13	-1.25	Kruppel-like factor 5 (intestinal)	MMP1	-17.72	-3.23	matrix metallopeptidase 1 (interstitial collagenase)
SAT1	-2.16	-2.16	spermidine/spermine N1-acetyltransferase 1	ETS1	-22.11	-1.74	v-ets erythroblastosis virus E26 oncogene homolog 1 (avian)
EMP3	-2.18	-1.50	epithelial membrane protein 3	C15orf48	-22.70	-1.34	chromosome 15 open reading frame 48

Supplementary Table 2. Primer Sequences Used For RT-PCR

Gene	Forward	Reverse
JAG2	GTGGATGTCGACCTTTGTGA	GGCAGTCGTCAATGTTCTCA
Notch3	ATGGTGGGAACTAAACACAGCT	ATGACCCTGGAGGAAGCACA
HES1	GTGCTGTCTGGATGCGGAGT	GAACACTCACACTCAAAGCCC
IHH	CCTGAACTCGCTGGCTATCT	AATACACCCAGTCAAAGCCG
SMO	GAATCGCTACCCTGCTGTTA	TGAGCAGGTGGAAGTAGGAG
GLI1	AGAGTCCAGGGGGTTACATA	CCTACCAGAGTCCCAAGTTT
FZD7	CCAACGGCCTGATGTACTTT	GCCATGCCGAAGAAGTAGAG
β-Catenin	GTATGAGTGGGAACAGGGATTT	CCTGGTCCTCGTCATTTAGC
NANOG	ACTGTCTCTCCTCTTCCTTCCT	AGAGTAAAGGCTGGGGTAGGTA
OCT4	GTGGAGGAAGCTGACAACAA	AGCAGCCTCAAAATCCTCTC
PTEN	GGACGAACTGGTGTAATGAT	CAGACCACAAACTGAGGATT
β –Actin	GGCATCCTCACCCTGAAGTA	GGGGTGTTGAAGGTCTCAAA
