# Supplementary figures and images for: Novel Gastric Cancer Stem Cell-Related Marker LINGO2 Is Associated with Cancer Cell Phenotype and Patient Outcome
Source: Int J Mol Sci. 2019 Jan 28;20(3):555. doi: 10.3390/ijms20030555 (PMC6387145; doi:10.3390/ijms20030555)

Supplementary Figure 2

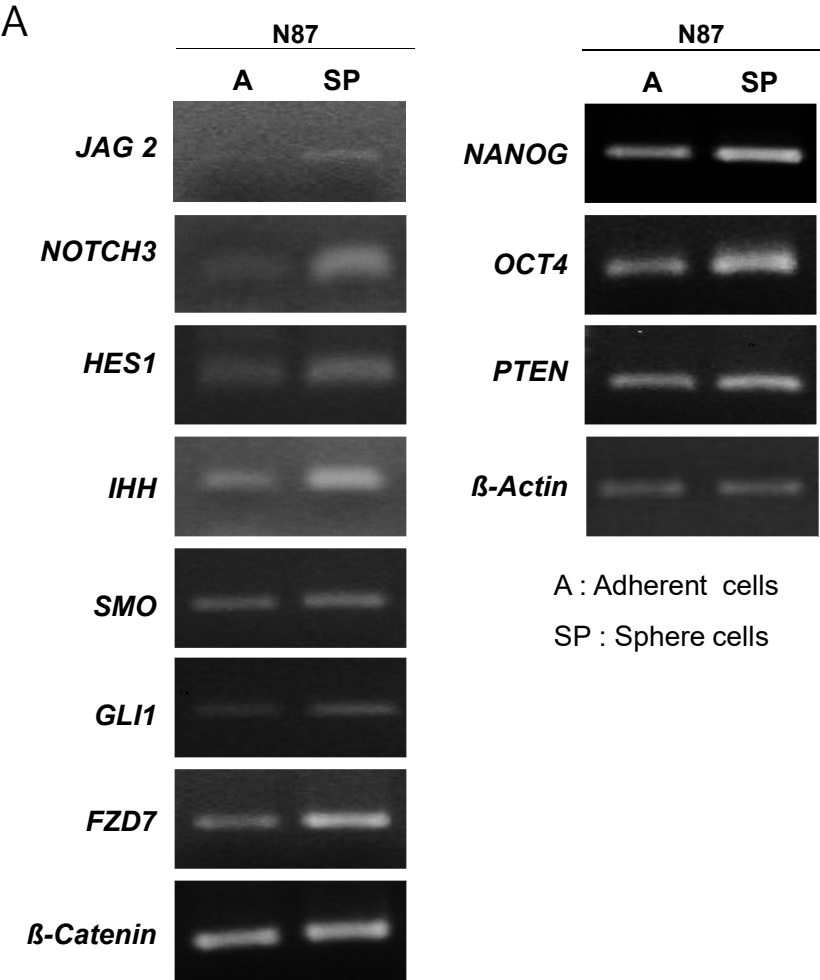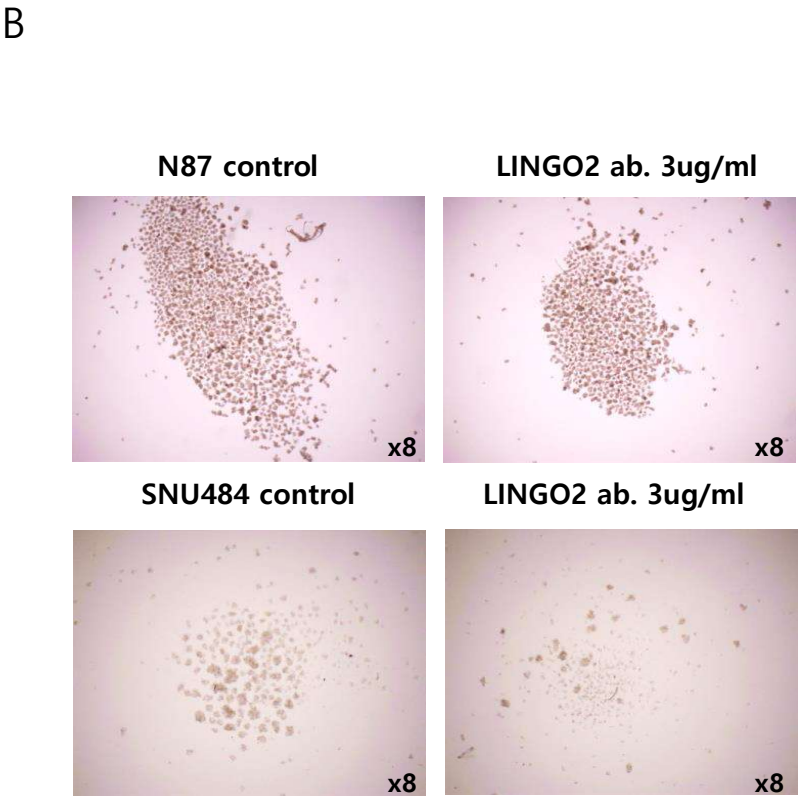

Supplementary Figure 3

A

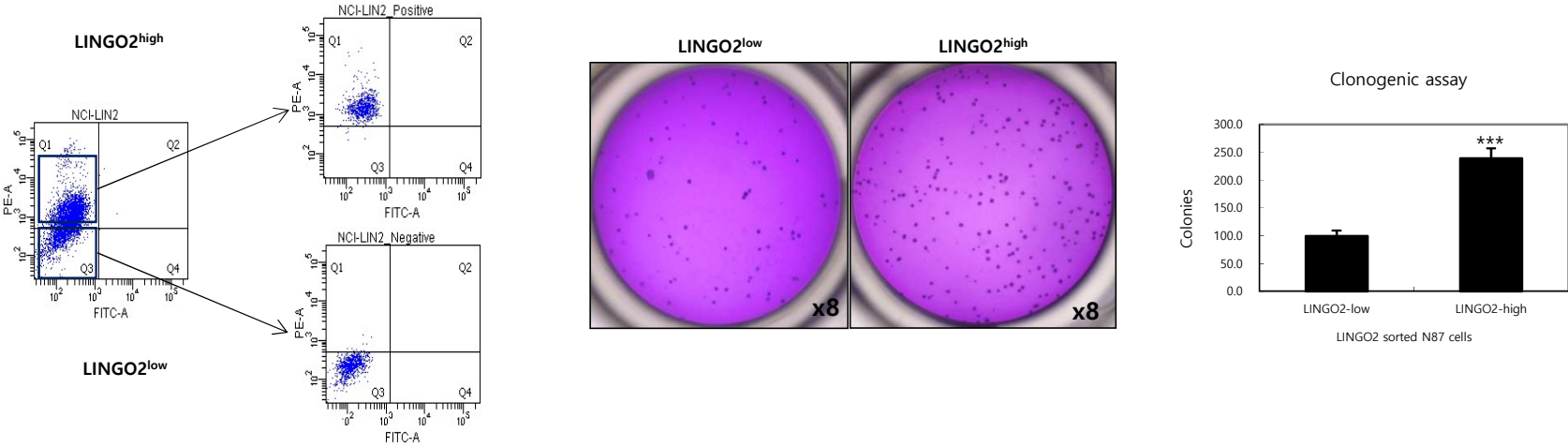

Supplementary Figure 4

A

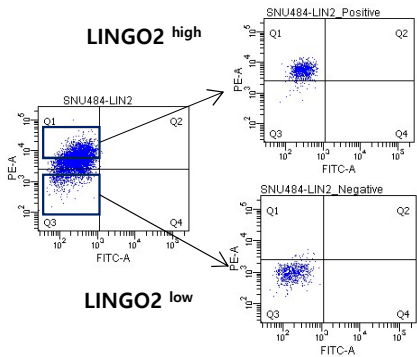

B

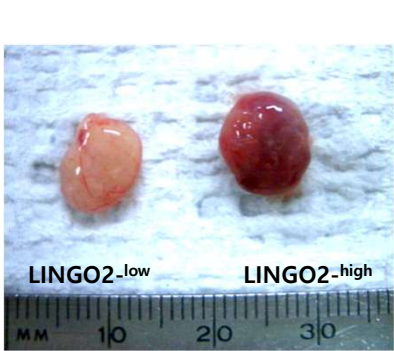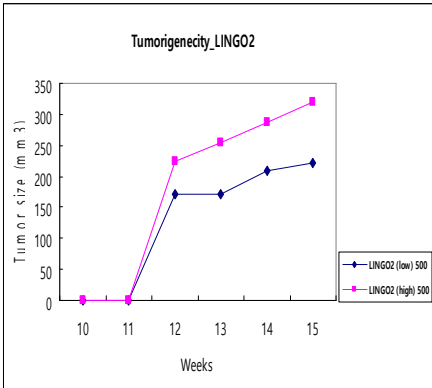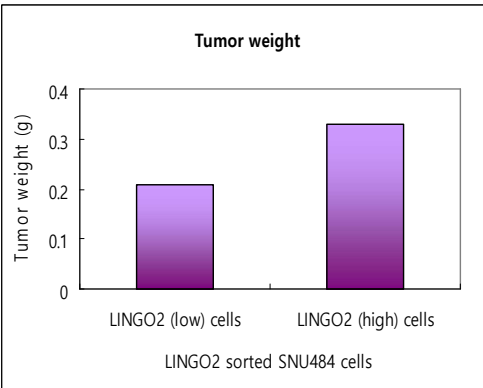

Supplement: Supplementary file 1 [file ijms-20-00555-s001.zip › supplementary/supplementary Figures.pdf]
